# The Implication of Reactive Oxygen Species and Antioxidants in Knee Osteoarthritis

**DOI:** 10.3390/antiox10060985

**Published:** 2021-06-21

**Authors:** Nicoleta Bianca Tudorachi, Eugenia Eftimie Totu, Adrian Fifere, Valeriu Ardeleanu, Veronica Mocanu, Cornelia Mircea, Ibrahim Isildak, Katarina Smilkov, Elena Mihaela Cărăuşu

**Affiliations:** 1 Faculty of Medicine, “Ovidius” University of Constanța, Mamaia Boulevard 124, 900527 Constanța, Romania; nicoletatudorachi@gmail.com (N.B.T.); valeriu.ardeleanu@gmail.com (V.A.); 2 Faculty of Applied Chemistry and Material Science, University Politehnica of Bucharest, 1–5 Polizu Street, 011061 Bucharest, Romania; 3 Centre of Advanced Research in Bionanoconjugates and Biopolymers Department, “Petru Poni” Institute of Macromolecular Chemistry, 41A Grigore Ghica Voda Alley, 700487 Iasi, Romania; 4 Faculty of Pharmacy, Grigore T. Popa University of Medicine and Pharmacy Iasi, 700115 Iasi, Romania; veronica.mocanu@gmail.com (V.M.); cornelia.mircea@umfiasi.ro (C.M.); 5 Faculty of Chemistry-Metallurgy, Department of Bioengineering, Yildiz Technical University, Istanbul 34220, Turkey; iisildak@gmail.com; 6 Faculty of Medical Sciences, Division of Pharmacy, Department of Applied Pharmacy, Goce Delcev University, Krste Misirkov Street, No. 10-A, 2000 Stip, North Macedonia; katarina.smilkov@ugd.edu.mk; 7 Faculty of Dental Medicine, “Grigore T. Popa” University of Medicine and Pharmacy, “Nicolae Leon” Building, 13 Grigore Ghica Street, 700259 Iasi, Romania; mihaelaelenacarausu@gmail.com

**Keywords:** oxidative stress, ROS, antioxidants, pathophysiology, KOA, cellular senescence, apoptosis

## Abstract

Knee osteoarthritis (KOA) is a chronic multifactorial pathology and a current and essential challenge for public health, with a negative impact on the geriatric patient’s quality of life. The pathophysiology is not fully known; therefore, no specific treatment has been found to date. The increase in the number of newly diagnosed cases of KOA is worrying, and it is essential to reduce the risk factors and detect those with a protective role in this context. The destructive effects of free radicals consist of the acceleration of chondrosenescence and apoptosis. Among other risk factors, the influence of redox imbalance on the homeostasis of the osteoarticular system is highlighted. The evolution of KOA can be correlated with oxidative stress markers or antioxidant status. These factors reveal the importance of maintaining a redox balance for the joints and the whole body’s health, emphasizing the importance of an individualized therapeutic approach based on antioxidant effects. This paper aims to present an updated picture of the implications of reactive oxygen species (ROS) in KOA from pathophysiological and biochemical perspectives, focusing on antioxidant systems that could establish the premises for appropriate treatment to restore the redox balance and improve the condition of patients with KOA.

## 1. Introduction

Knee osteoarthritis (KOA) is known as a chronic degenerative disease and a significant public health issue worldwide; the economic burden associated with it rises with population aging in most countries [1]. It is one of the prevalent causes of disability, extensively affecting patients’ quality of life [2]. Whereas KOA used to be considered a geriatric pathology, lately, it has been recorded among ever-younger patients [3]. Moreover, KOA prevalence has increased considerably in older adults (persons aged 60 and over) [4]. During the last decade, an increased number of newly diagnosed cases has been recorded [5]. In addition, by 2030, the number of arthroplasties is estimated to increase by 276% [6,7], while the number of specialty consults is expected to rise by 15.7% in 2032, compared to 2012 [8].

The role of oxidative stress in KOA physiopathology is still a topic of analysis. The effects of free radicals on cartilage components involve cellular senescence and apoptosis, while their existence is conditioned by endogenous or exogenous factors characterized by specific chemical and physical parameters. The most important chemical parameters determining or marking a particular physiological process are pH and the redox state. These parameters have different values as a function of the analyzed organ or tissue within the body, the living conditions, or the existence of certain pathologies [9,10].

Along with other risk factors, all these alterations favor the onset and progression of KOA and impose a series of blood tests enabling the identification of free radicals and serum antioxidants levels for tailoring a personalized therapeutic approach that could include sources of antioxidants. In this context, it is essential to develop appropriate strategies and identify the appropriate prevention solutions by discovering and reducing alterable risk factors [9]. For instance, weight control, active life, and a diet rich in antioxidants, as well as antioxidant therapies, are essential pillars of KOA prevention. The aim of this review is to highlight the influence of ROS and antioxidants in KOA among older adults, analyzed from pathophysiological and biochemical perspectives. The authors performed an electronic literature survey in the PubMed, EMBASE, Web of Science, ScienceDirect, and Google Scholar databases using keyword search terms. The investigation was completed with a manual search. In this review, only published English-language papers were considered. The present work includes cohort studies, randomized, double-blind, placebo-controlled clinical trials, meta-analyses, and reviewed studies. 

## 2. ROS and Antioxidants

ROS comprise species with unpaired electrons, also called paramagnetic, such as free radicals, diamagnetic species with oxidant properties, or chemical species that (due to their higher energetic state) deactivate through an oxidative process. The most reactive ROS are those belonging to the class of free radicals, such as hydroxyl (HO•), alkoxide (RO•), superoxide (O_2_^•−^), and peroxyl (ROO•). The species with paired electrons represent another ROS class; often, they are the product of the reactions of species from the first category, namely hydrogen peroxide (H_2_O_2_), organic peroxide, ozone (O_3_), or charged species, such as singlet oxygen (^1^O_2_) and excited carbonyl (RCH = O*) [11]. Oxygen-free radicals are produced as a result of exposure to polluted air, ultraviolet radiation, tobacco, alcohol, and drugs. The primary endogenous sources are cellular enzymes, myeloperoxidases (MPO), lipoxygenases, nicotinamide adenine dinucleotide phosphate (NADPH) oxidase (NOX) [12], and xanthine oxidase [13].

The oxidative interface is the boundary between ROS and the signaling molecules they activate; it describes how ROS directly activate oxidative stress-responsive pathways [14,15].

The oxidative str*ess* could be defined as an imbalance between the production and ROS accumulation at the cell level and cells’ capacity to inactivate them by using enzymatic reactions or other redox reactions [10,16]. Oxidative stress is the result of excessive production of ROS, which exceeds the capacity of the cellular antioxidant defense system to effectively remove them from the cells [14].

Antioxidants may be defined as reducing substances that oxidize, thus inhibiting another substrate’s oxidation. Through a more complex mechanism, they have a kinetic effect of slowing down the oxidation reaction [17]. 

Superoxide anion is considered the primary free radical molecule formed in living organisms through chemical and biochemical reactions. Molecular oxygen (O_2_) is converted into water at a mitochondrial level in the electron transport chain [18]. A small part of O_2_ is transformed into O_2_^•−^, which further generates different reactive species, such as HO• and H_2_O_2_; they produce other ROS by interacting with various organic molecules within the body. Frequently, superoxide radicals do not react directly with nucleic acids, carbohydrates, or polypeptides, determining the emergence of other reactive species, leading to oxidative processes [18,19]. In the human body, superoxide free radicals are inactivated under the actions of the superoxide dismutase enzyme (SOD), being converted into H_2_O_2_ within a two-phase chemical process [20,21].

Hydrogen peroxide is a regular metabolic product in the human body. When in higher quantities, it determines the production of HO• through Fenton-type reactions.

It is impossible to determine H_2_O_2_ concentrations accurately because of the many factors that cause variations. Still, the values in plasmatic blood are around 1–5 µM [22]. Low concentrations of H_2_O_2_ are part of normal, adaptive processes as a response to stress. By contrast, high concentrations are included in oxidative stress, determining inflammations, senescence, and even cellular apoptosis. Moreover, H_2_O_2_ could be inactivated in the human body by endogenous antioxidants, such as catalase (CAT) and glutathione (GSH), in the presence of GSH peroxidase (GPx) [20]. 

Hydroxyl radical represents one of the most important free radicals belonging to ROS species. This radical is extremely reactive, and it is the main cause of lipid peroxidation. One of the main pathways to generate this radical within the human body is represented by Fenton reactions in the presence of metals [23]. 

ROS are involved in several physiological cellular functions, serving as important cellular messengers in normal signal transduction, gene regulation, and cell cycling [24]. ROS-induced cell signaling involves two general mechanisms: alterations in the intracellular redox state and oxidative modification of proteins [25]. Cells respond to ROS in different ways depending on the redox status. Under physiological conditions, increased production of ROS triggers cellular defense, and the mechanism against oxidative stress is activated by effectively removing ROS molecules from the cell [26]. 

**Figure 1:**
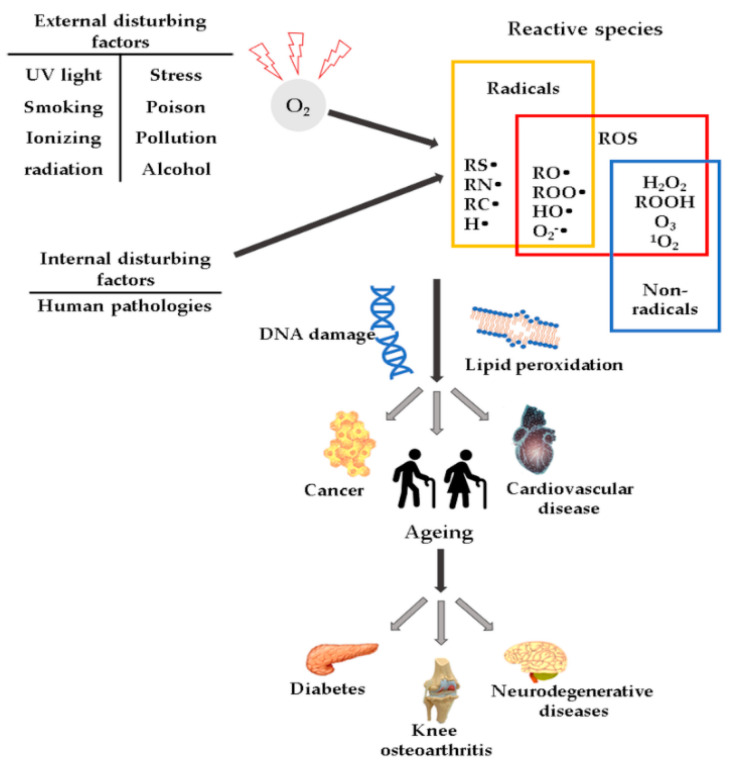
Schematic representation of ROS and their effects.

High ROS concentrations may influence the induction or aggravation of several pathologies (Figure 1), such as hypertension [27], cardiovascular diseases, diabetes mellitus (DM) and its complications [28], metabolic syndrome [28,29,30], cancer [31], neurodegenerative diseases [32], or DNA mutations [33]. 

### 2.1. Antioxidants

Antioxidants comprise several species that play a vital role in the suppression and prevention of free radicals, as well as in the neutralization of molecules with the potential of becoming free radicals. The human body controls the oxidative level through a series of enzymatic and nonenzymatic antioxidants [34]. 

#### 2.1.1. Enzymatic Antioxidants

The enzymatic cellular antioxidant defense system includes various classes of enzymes, such as SOD, CAT, and GPx. Enzymes act specifically by eliminating ROS or synergically by catalyzing the regeneration of other antioxidants [35]. 

SOD is a class of enzymes comprising three isoenzymes with localization in extracellular, mitochondrial, or cytoplasmatic components. They have been found in reduced amounts in the case of KOA, especially the type 2 and 3 isoenzymes [35,36]. They have a role in converting O_2_^•−^ in O_2_ and H_2_O_2_, while higher serum levels of SOD are associated with lower general mortality rate among the feminine geriatric population [37]. 

CAT is an antioxidant enzyme with different localizations and a role in catalyzing H_2_O_2_ dismutation to water and oxygen. CAT plays an important role in H_2_O_2_ metabolism as a key regulator in maintaining cellular redox homeostasis [38]. Studies on lab animals have highlighted the antioxidant function of CAT, which increased the life span and reduced aging processes induced by oxidative stress [39]. Paradoxically, in the case of patients with KOA, the CAT serum level is high. Upon correlating all evidence, it may be stated that this is due to the compensating protective role of CAT [40]. Collins et al. [39] confirmed these effects of CAT at the level of human chondrocytes and lab animal cartilages. Their studies presented a CAT-induced mitochondrial expression by mitigating ROS-induced oxidation and hyperoxidation of peroxiredoxins. The targeted expression of mitochondrial CAT has a role in decreasing ROS, as it helps cell survival and reduces the severity of arthritic symptoms. CAT-induced effects included reduced severity of degenerative alterations and increased survival rate of chondrocytes. 

GPx is an enzyme with an important role in the reduction of H_2_O_2_ and lipid peroxides [41]. This enzyme has four isoforms with different locations. Thus, classic GPx is found in most cells of the body; the gastrointestinal isoenzyme (GI-GPx) is located in the gastrointestinal tract and forms a barrier against hydroperoxides derived from the diet. Plasma GPx is located in the plasma, and phospholipid hydroperoxide GPx is located in the mitochondria and is considered a universal antioxidant enzyme that acts on lipids and lipoproteins damaged by free radicals [42]. *Prx* enzymes have an antioxidant role at the intracellular level, also regulating redox signaling events. Prx enzymes are present in all types of tissues, including cartilage, where they act as redox signaling agents for target proteins containing thiol groups, with high activity against H_2_O_2_ [43]. Prx may disrupt cellular signaling in older adults because of the increased sensitivity to hyperoxidation, inhibiting their function by increasing intracellular ROS levels. In older adults, the Prx isoforms 1–3 have higher activity in chondrocytes under conditions of oxidative stress, compared to younger people. These imbalances suggest a phenotype typical of geriatric patients’ tissues [39].

#### 2.1.2. Nonenzymatic Antioxidants Comprise Compounds such as GSH, Carotenoids, Vitamin C, Vitamin E, or Coenzyme Q10

GSH- is an antioxidant of endogenous source; it is a tripeptide comprising amino acids such as glycine, L-glutamic acid, or L-cysteine [44]. It is found intracellularly in mitochondria and peroxisomes and has a role in preventing ROS-induced cellular degradation, which it can eliminate through enzymatic or nonenzymatic reactions. These reactions involve GPx, glutathione-S transferase (GST), glutathione reductase (GR), and modulations of the tertiary protein structure. In the cytoplasm, GSH is coupled to SOD and ensures the protection of subcellular structures against oxidative stress. The modifications of GSH concentrations lead to cell senescence and apoptosis through alterations of their proliferation and the detoxifying and enzymatic transcription processes [45,46]. 

Carotenoids are terpenoid pigments and are found in vegetables, fruits, shellfish, eggs, and meat. They are detected in the human body, and the most common isoforms are β-carotene, α-carotene, lutein, or lycopene [47]. Carotenoids have antioxidant effects and inhibit the activation of nuclear factor kappa-light-chain enhancer of activated B cells (NF-kB), interleukin (IL) -6, and tumor necrosis factor-alpha (TNF-α). β-Carotene reacts with ROO•, OH•, and O_2_^•−^ [48]. Vitamin C is an important nonenzymatic antioxidant that helps reduce free radical damage. Recent studies have shown that vitamin C can have beneficial effects in patients with early KOA [49]. There are two isoforms of vitamin C transporters that are sodium dependent. At the level of articular cartilages, transporter 2 is mainly expressed, which is significantly altered in KOA grade III, compared to KOA grade I [50]. Vitamin C also acts as a biological modulator in immune cells; it is involved in the synthesis of collagen and catecholamines, and there is evidence that the administration of high doses of vitamin C improves body function and the survival of critically ill patients [51]. Vitamin E is a powerful antioxidant that has a potential preventive or curative role in KOA, due to its antioxidant and anti-inflammatory properties [52]. The antioxidant activity of vitamin E is due to the release of hydrogen from the hydroxyl group on the chromanol ring [53]. Vitamin E is a group of compounds, including tocopherols and tocotrienols, with alpha-, beta-, gamma-, and delta isoforms [54], of which alpha-tocopherol is the most important one [55]. Alpha-tocopherol can have both antioxidant and pro-oxidant effects when present in high doses by acting on vitamin K at the bone level and could block other types of vitamin E isomers [55]. Previous studies have demonstrated that the antioxidant activity of tocotrienol is superior to that of alpha-tocopherol [56,57]. Coenzyme Q10 is a coenzyme for mitochondrial enzymes involved in the electron transport chain and has a role in adenosine triphosphate (ATP) synthesis. It has an antioxidant effect by inhibiting the peroxidation of plasma lipoproteins and lipids of cell membranes, but its anti-inflammatory effects have also been highlighted. The antioxidative and anti-inflammatory effects have been demonstrated in animal studies when it was observed that the oxidative stress was reduced and the histological score of the cartilages affected by KOA was improved [58,59]. 

## 3. ROS Implication in KOA Pathogenesis 

ROS are considered key etiological factors involved in the pathogenesis of many age-related diseases [34]. A typical geriatric patient is characterized by a fragile state with a low functional reserve; thus, such patients are more prone to oxidative stress, stress factors, and inflammation [60]. The impact of aging on the cartilage structure is also highlighted by the macroscopic appearance of the tissue among subjects of various ages. Although still intact and smooth in elderly patients, the cartilage is thin and brownish [61]. The accumulation of degradation products may cause this appearance and the alterations recorded in chondrocytes functions [62]. The exogenous and endogenous factors to which the human body is exposed over time act on all these processes, leading to destructive effects that may or may not be remedied [63]. 

### 3.1. ROS, Aging, and Inflammation in KOA Pathogenesis

ROS could affect the musculoskeletal system, including multiple cellular structures at the bone level [64]. 

**Figure 2:**
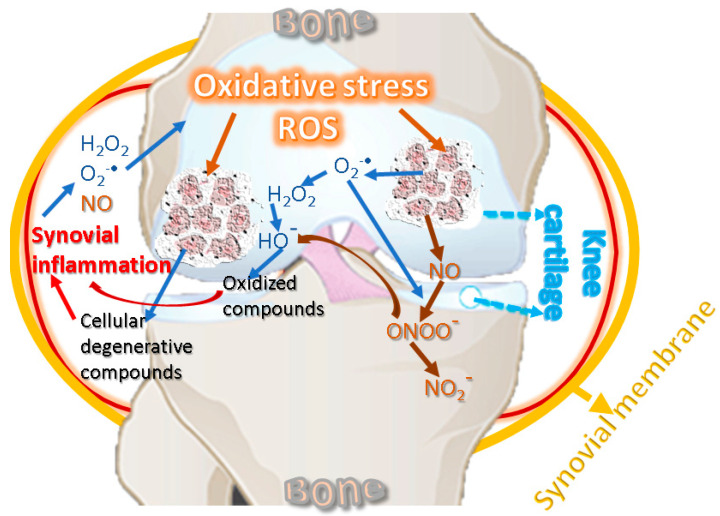
Oxidative stress and inflammation reciprocal cause-and-effect cycle.

The pathophysiology of KOA involves inflammatory and degenerative processes related to oxidative stress [65]. Low-grade systemic inflammation can be caused by aging [66] and can affect the knee joint by supporting local inflammation [67]. Inflammation and oxidative stress are interdependent and activate the signal transduction pathways in cartilage, producing a phenotypic change characterized by the inability of KOA chondrocytes to maintain tissue homeostasis [68,69]. Inflammatory changes decrease the level of antioxidant enzymes in biological fluids and cartilage and increase the level of oxidative agents, affecting proteins with a role in structural strength [24]. Figure 2 suggests the interdependence relationship between oxidative stress and inflammation, showing the cycling sequence, in which the oxidative stress and synovial inflammation are intensifying each other. 

ROS are also produced by the NOX group [24], which comprises several forms, such as NOX1, NOX2, NOX3, NOX4, and NOX 5 [70]. The NOX-mediated ROS signaling underscores an isoform-specific to this oxidase, namely NOX4, targeting chondrocytes with a role in the standard final pathway [71]. ROS are key factors in the chondrocytes apoptosis because these could induce the appearance of the others. Under normal conditions, ROS products are used in joint chondrocytes to maintain cartilage homeostasis, actively participating in intracellular signaling. For example, in human chondrocytes, NOX produces O_2_^•−^, which further generates H_2_O_2_ and HO• radicals [36,72,73]. Under pathological conditions, chondrocytes produce an important amount of reactive species [74], and NOX induces ROS synthesis in the synovial fluid, leading to oxidative stress, thus contributing to cartilage degradation [75]. The main ROS affecting cartilage detected in chondrocytes are H_2_O_2_ and peroxynitrite. In chondrocytes, ferrous ion (Fe^2+^) and H_2_O_2_ produce HO•, which reacts with the unsaturated fatty acids of membrane lipids, thus forming lipid radicals RO• and ROO•. ROS are neutralized by elimination systems, namely, SOD, CAT, GPx, GR, and GSH, but limited antioxidant capacity has been reported in KOA human cartilage and experimental animal models. Therefore, oxidative stress leads to membrane and nucleic acid damage, as well as to the breakdown of extracellular components, including proteoglycans, collagens, and cartilage destruction [20,23,45]. Similarly, in vitro studies performed on bovine chondrocytes suggested that O_2_^•−^ is involved in load-induced KOA development [76]. Chondrocytes are cells of cartilages, avascular, and innervation-free tissues that allow oxygen to diffuse into superficial areas producing ROS [77]. In pathologies such as KOA that combine mechanical and inflammatory stress, as well as variations in oxygen pressure, chondrocytes produce much higher levels of ROS through NADPH [78]. ROS shows inhibitory effects in cartilage synthesis by inhibiting the synthesis of proteoglycans by NO in the superficial and deep layers of cartilage [79], and H_2_O_2_ inhibits their synthesis inside chondrocytes by forming ATP and suppressing mitochondrial oxidative phosphorylation [80]. 

**Figure 3:**
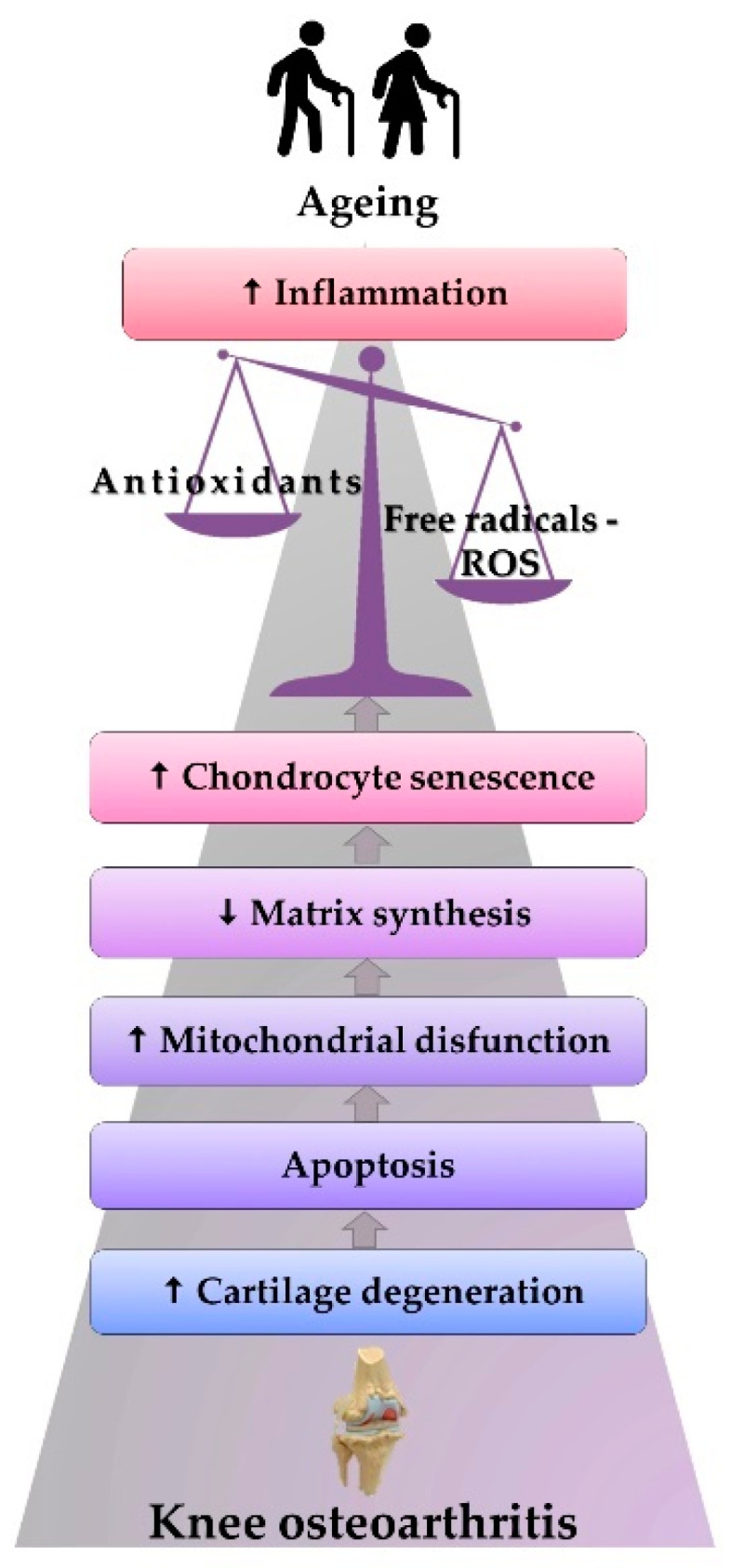
The physiopathological mechanism of ROS-induced KOA.

Excess O_2_^•−^ in mechanical overloading conditions was confirmed indirectly by biochemical analyses (flow cytometry) of cartilage taken from patients with KOA. Thus, the correlation between decreased SOD concentration and cartilage degeneration in the progression of KOA was highlighted [81]. These observations correlate well with the general idea that antioxidant enzymes, such as SOD, CAT, and GPx, have low levels in patients with KOA, suggesting the critical role that ROS and oxidative stress play in this pathology [82,83,84]. Therefore, reduced quantities of antioxidant enzymes determine high oxidative and metabolic stress for cartilages, at a level where, besides all the molecular and functional alterations, changes are also noted in the ratio between collagen and proteoglycans, thus favoring the onset of degenerative changes [85,86]. In addition, with aging, chondrocytes become more sensitive to the action of ROS, which is associated with an increase in inflammation mediators at serum level, impacting the extracellular matrix, producing chondrocytes senescence and apoptosis [85], and causing joint cartilage degeneration (Figure 3), subchondral sclerosis, and damage of meniscal and ligament structures [87]. The presence of lipid peroxidation products in the case of KOA was correlated with the action of ROS. Thus, the level of oxidized low-density lipoprotein (ox-LDL), nitrated derivatives, nitrotyrosine, or nitrite in the cartilage and the biological fluids evidence the ROS implication [88,89]. The KOA pathogenesis depends extensively on the infrapatellar pad and menisci. It has been reported that in the case of KOA, the meniscal cells produce increased levels of inducible nitric oxide synthases (iNOS) and NO [90], while the KOA progression is favored by the infrapatellar fat pad known as a source of inflammatory mediators [91]. 

Figure 3 features KOA stages among older adults: aging is correlated with the effects generated by the increase in the inflammatory status and oxidative stress. Such stress causes the onset of mitochondrial dysfunctions and a decrease in matrix synthesis. All the alterations accelerate chondrocytes’ senescence, leading to apoptosis, cartilage degeneration, and the beginning of KOA.

The susceptibility to changes generated by ROS is highest at the superficial layer of the knee cartilage, which has direct contact with the synovial fluid containing free radicals and inflammatory cells. The long-term action of ROS, as well as the intense activity of inflammatory and mechanical factors, modifies the structure of collagen fibers and causes the thinning of the proteoglycans layer. Moreover, functional alterations affect the deeper cartilage areas [86]. As a result, the main symptoms of KOA consist in pain and functional impotence [92], in association with radiological changes [93]. It is worth mentioning here that ROS have important implications in the induction of a senescence secretory phenotype, thus resulting in supporting the inflammatory status by increasing the production of IL-1 and IL-6, or matrix metalloproteinases (MMPs) [85]. 

Oxidative stress also induces the activation of the NF-κB pathway and MMPs overproduction, thus triggering DNA damage and cell senescence [68,69,94]. Several studies demonstrated the pivotal role of NF-κB in KOA pathogenesis. The family of transcription factors NF-κB have a central role not only in the proinflammatory stress-related responses of chondrocytes but also in the control of their differentiation program. NF-κB is activated by the signalosome complex, a molecular switch controlled by two pivotal serine-threonine kinases that can remove NF-κB inhibitors, which block NF-κB transcriptional activities: inhibitors of nuclear factor kappa-B kinase (IKK) β and IKKα [66,69]. Abnormal NF-κB activation produced the loss of the growth-arrested state of articular chondrocytes, being accompanied by the production of procatabolic mediators. Among these mediators are included the aggrecanases and MMPs, which induce cartilage degradation, as well as the proinflammatory cytokines. Further, continued NF-κB activation results in the overexpression and activation of other regulatory transcription factors, perpetuating KOA disease via modulation of inflammatory and catabolic mediators, and linking hypertrophic-like conversion with inflammation. Together, these signaling networks collaborate to activate MMP13 expression and activity and facilitate the progression of normal articular chondrocytes to a hypertrophic-like KOA phenotype, thereby also contributing to KOA onset and/or progression [68,69]. 

### 3.2. ROS, Damage of Genomic and Mitochondrial DNA, and Cell Senescence

Oxidative stress also affects DNA: it accelerates senescence and stimulates the activation of molecule cascades, generating the onset of cellular apoptosis. Mitochondrial DNA (mtDNA) haplogroups, which mediate energy production, cell growth, and cell signaling for molecular pathways [95], have also been associated with specific population groups with KOA radiological changes, for instance haplogroup H, or lower level of free radicals and proapoptotic gene expression at the mitochondrial level and thus a lower grade of cellular apoptosis, as in the case of haplogroup J [96]. These different ways of influencing certain pathologies are explained by the fact that haplogroups have different rates of ROS and energy production. For example, in haplogroup H, high amounts of energy and ATP are produced, while an excess release of ROS and cellular degenerescence occur [95]. 

In KOA, catabolic processes are accentuated by the combined action of oxidative stress and proinflammatory state, and mechanical factors, entailing high ROS levels and low amounts of antioxidants [97,98]. The low concentrations of antioxidants amplify ROS signaling and favor mitochondrial dysfunction in chondrocytes [99]. These alterations at the mitochondrial level occurred because of the ATP-altered synthesis and decreased electrons within the mitochondrial electron transport chain [100]. Increased ROS concentration and aging are also determined by an interruption of cell signaling at the mitochondrial level, accompanied by the production of specific cell dysfunctions and sometimes tissue dysfunctions [101]. When homeostasis is present at the level of bone tissues, the differentiation of osteoblasts and bone formation is ensured by mitochondria. The increase in ATP production and mitochondrial stress induced the activity of SOD isoform 2. This antioxidant enzyme has a role in reducing oxidative stress, improving osteoblast differentiation, bone remodeling, and mitochondrial stress regulation [102]. Such a statement is based on the hypothesis that there is a direct effect of inflammatory molecules on DNA mutations [103]. Persistent mtDNA damage is recognized as a hallmark of chronic degenerative diseases [68]. The mtDNA damage is considered responsible for the senescence of OA chondrocytes, which may be caused by an increase in mitochondrial ROS production and shortening of telomeres, which is dependent on stress factors [104]. Moreover, ROS induces matrix loss, premature senescence, mitochondrial dysfunction, apoptosis of chondrocytes, mesenchymal stem cells [105], and subchondral bone mass loss [106]. The inhibitory effect of oxidative stress in bone remodeling was revealed by the low serum level of certain antioxidants and the increase in the concentration of lipid peroxides; these specific serum levels highlighted a disturbance of the redox balance, with harmful effects of ROS on the DNA [107]. The resulted dysfunctions and the generation of cytotoxicity lead to lower levels of antioxidant enzymes in biological fluids and cartilages, as well as an increase in oxidative markers affecting the proteins that ensure structural resistance [24]. Therefore, similarly to other pathologies [108], in KOA, the correlation with molecular biomarkers is important [24].

## 4. Markers of Oxidative Stress in Older Adults with KOA

Biomarkers must meet certain criteria to be used in medical practice. They must allow easy sampling, and they should be reproducible. At the same time, it should have diagnostic and prognostic specificity and be able to correlate with the severity of the pathology in order to evaluate the effectiveness of the treatment [109]. Although multiple determinations of oxidative stress markers and antioxidant status are performed, dosing on large groups of patients is required for clinical and diagnostic validation. Because oxidative stress characterizes several pathologies [110,111,112], it is vital to perform a targeted selection of biomarkers in accordance with each disease [109,113]. The biomarkers for oxidative stress or the antioxidant markers can be measured in blood, urine, or saliva samples [114], as well as in the expired air by different analytical methods [115]. 

Lipid peroxidation involves changes in the composition and structure of cells, with adverse effects on their function [116]. The lipid membrane is affected by oxidative stress related to aging. There are known two types of lipid peroxides: endoperoxides and hydroperoxides. They generate aldehyde products, such as malondialdehyde (MDA). Such products have destructive effects on the extracellular matrix, cellular components, and implicitly on the proteins and DNA [117], also producing other metabolites, such as 4-hydroxynonenal (4-HNE), F2-isoprostanes (F2-IsoPs), acrolein, hexanoyl-lysine, or ox-LDL [118]. After local injection of 4-HNE to animals, macroscopic and microscopic changes in articular cartilage and increased concentrations of inflammatory markers were observed [119]. The determination of lipid peroxidation markers can be performed using chromatography, mass spectrometry (MS), or spectrophotometric absorption analysis. These are sensitive procedures that ensure the monitoring of multiple reactions, maintaining the stability of the analyte and of certain isotope species [120]. 

MDA is a toxic aldehyde and is used as a biomarker of lipid peroxidation [121]. There are several extremely precise methods of analysis, such as reverse-phase high-performance liquid chromatography and modified 2-thiobarbituric acid spectrophotometric method [120,122]. The dosage can be performed in human plasma by 2,4-dinitrophenylhydrazine derivatization, and the results can be used to assess the carbonylation of proteins, or the oxidation of lipids and proteins [123]. MDA presents high values in KOA, having effects on the degradation and oxidation of collagen in cartilage. Thus, Gavriilidis et al. [99] observed a significant 6.5% increase in MDA values in patients with KOA, compared to a control group. Elevated MDA levels have also been associated with other conditions, such as lung diseases [124]. Comparative analysis of certain free radical types present in the plasma and the synovial fluid from KOA patients and a control group has shown significantly higher plasma values for the test group. Among patients with KOA, MDA was recorded in substantially higher amounts in the plasma and synovial fluid. In addition, severe changes in grades III and IV of KOA, according to the radiological Kellgren–Lawrence classification system (KL), were correlated with high serum levels of free radicals and MDA [125]. According to the researchers, relatively low MDA levels in certain groups of the geriatric population could indicate the presence of a genetic component with a protective effect against oxidative stress [126]. Regarding the administration of certain exogenous antioxidants, the concentration of MDA can be reduced by curcuma [127], turmeric [128], and ginger extracts [129].

MPO is a proinflammatory hem peroxidase expressed in lymphocytes, leukocytes, or neutrophils, and is released following cell degranulation. It also generates ROS during phagocytosis in response to microbial attack. However, in uncontrolled degranulation processes, it can cause tissue damage and aggravate inflammation even in the absence of infectious processes [130]. It can be used as an indirect biomarker measurement or for assessing MPO-specific biomarkers, such as chlorotyrosine, glutathione sulfonamide, or chlorinated lipids [131]. Geriatric pathologies that can be associated with high concentrations of MPO comprise KOA [132], DM, liver [130], or cardiovascular diseases [133]. 

Isoprostanes (IsoPs) are generated by the peroxidation of polyunsaturated fatty acids. The species F2-IsoPs are the most common, derived from arachidonic acid [134], and can be used as a mediator of oxidative stress or a nonenzymatic biomarker of endogenous lipid peroxidation [135,136]. Elevated concentrations of IsoPs were detected in serum, synovial fluid, and synovial membrane biopsy in patients with KOA or meniscal and cruciate ligament lesions [137]. The determinations could be performed from plasma, saliva [138], cerebrospinal fluid samples [136], or various tissue types, by gas chromatography-mass spectrometry (GC-MS) methods [138]. Decreased F2-IsoPs levels are associated with vitamin C administration [139] and with the consumption of antioxidant-rich fruits [140]. 

Protein carbonyl can be used as a marker of protein oxidation, the carbonylation process being irreversible and generating changes in DNA and enzymatic activities of the transcription factors [141]. The serum concentration of carbonylated proteins correlates negatively with albumin levels, an extracellular molecule known as positive acute-phase protein. Such correlation reflects the fact that a low level of serum albumin signals the presence of inflammation and oxidative stress [142]. Elevated carbonyl protein levels have been detected in multiple pathologies, such as DM [143], rheumatoid arthritis [144], chronic renal failure, or sepsis [145]. 

ROS-induced gene changes are premutagenic lesions, and guanine has the most remarkable predisposition to genome oxidation. Oxidation of the guanine base results in 7,8-dihydro-8-oxoguanine, which is considered a biomarker of oxidative DNA damage [146]. DNA mutations, with their effects on proteins and gene expression, have been considered one of the main causes of aging. These changes are associated with decreased B cell function [147], as seen in the cartilage affected by OA [148], at the neuronal level in the elderly [149], or in skeletal muscle, where approximately 13 somatic mutations per genome were recorded annually [150]. However, there is no direct evidence for attributing the aging process to DNA mutations [151].

Excessive ROS causes the oxidation of DNA, and the oxidized nucleosides are excreted in urine, so that the overall rate of DNA oxidation can be obtained noninvasively [152]. Measurements can also be made from peripheral blood, but this type of sampling has some drawbacks, for example, a longer working time, or possible damage to samples when they are improperly stored. New technologies have allowed the development of investigation methods that enable capillary blood dosing [153]. 

In medical practice, the analysis of total antioxidative capacity (TAC) or measuring its enzymatic or nonenzymatic markers is commonly used [154]. The assessment of the redox status can also include antioxidant markers that may be enzymatic, such as SOD, CAT, GPx, and TAC [155], or nonenzymatic, such as coenzyme Q10, GSH, retinoids, transferrin, ceruloplasmin, carotenoids, vitamin E, vitamin C, albumin, and uric acid [156]. 

TAC is a biomarker used to assess the body’s antioxidant potential [157]. However, since the enzymatic activities are excluded and thus the determination is only partial, the term nonenzymatic antioxidant capacity (NEAC) could be more appropriate [158]. Its determination is of real interest in medical practice; hence, several investigation methods have been developed [159]. The analysis can be performed from different types of biological fluids, such as peripheral blood, saliva, or urine. Because existing pathologies may influence the respiratory or urinary tract values, the analysis of urine and saliva should consider the influencing factors [157]. An optimal level of NEAC has been correlated with decreased risk of coronary events [160,161] and reduced degenerative changes in knee cartilage [162]. 

SOD can be used as a marker of antioxidant enzyme capacity. According to Koike et al. [163], this enzyme correlated with low synovial fluid levels in patients with KOA. These observations could indicate that, as SOD activity is not influenced by age, its corrections at the joint level could slow OA progression. During their investigations, Fernandez-Moreno et al. [154] found elevated SOD2 values in KL IV KOA cases. The researchers explained the findings through a compensatory mechanism of the body to cope with increased ROS concentrations. These results highlight the importance of correlating the ROS concentrations, determined using oxidative stress markers, with TAC, the clinical applicability of this assessment being of real interest for KOA in geriatric patients. 

CAT is an enzyme with an important antioxidant role, whose benefits in chondrocytes have been proven by lowering ROS levels, reducing TNF-α-induced apoptosis, and physiological remodeling of cartilage [164]. 

GPx is an enzyme that can reduce large amounts of hydroperoxide radical. GPx serum levels are measured to assess the body’s oxidative state [165]. KOA is correlated with low levels of GPx, which could be a side effect induced by high doses of nonsteroidal anti-inflammatory drugs [166]. In addition, the GSH/glutathione disulfide (GSSG) ratio allows the oxidative stress evaluation [167]. 

Research in mice to evaluate cartilage changes in OA using equilibrium partitioning of an ionic contrast agent via microfocal computed tomography, in correlation with ROS quantification and characterization by in vivo fluorescence imaging, revealed the presence of higher ROS amounts in the first day after experimentally inducing KOA [168]. The low level of antioxidant enzymes, especially SOD and GPx, was correlated by Paździor et al. [169] with KOA imaging changes, which were observed using conventional radiology, ultrasounds, or computed tomography, when investigating older female patients. 

S-glutathionylated proteins are oxidized proteins that characterize chronic inflammation and induce metabolic stress changes in monocytes and macrophages. They are also associated with aging, DM, cancer, or heart diseases [170]. 

Nonenzymatic markers have also been studied in the context of KOA: vitamin E, vitamin C, and coenzyme Q10. For instance, vitamin E is a powerful antioxidant of plant origin [55]. Lower concentrations of vitamin E in serum or synovial fluid were detected in patients with KOA, compared to healthy persons. Vitamin E is thought to relieve oxidative stress and joint inflammation by slowing KOA progression [52]. In a prospective longitudinal observational cohort study, the increased level of serum vitamin C was associated with better functional capacity and less pain in the knee joint [171]. Vitamins E and C, MDA, and 8-hydroxy-2-deoxyguanosine-marker of oxidized DNA can be used as biomarkers in other pathologies, other than KOA, when low concentrations of vitamin E and C and high levels of MDA and 8-hydroxy-2-deoxyguanosine were detected [114]. Objectifying the effects of vitamin E in KOA, through tests performed on lab animals, consisted in decreasing TNF-α and IL-6 [172]. In addition, other inflammatory markers within KOA are represented by TNF-α and IL-1β, IL-6, IL-15, IL-17, and IL-18, with a consecutively increasing effect of MMPs [173,174]. An inflammatory process lowers the concentration of antioxidants and increases the oxidative stress. At the same time, such alterations result in a decrease in type II collagen synthesis and in the acceleration of chondrocytes senescence. In these processes, specific degradation products induce changes in the cartilage properties, with excess production of MMPs and cytokines, thus leading to degenerative changes [173,175]. 

Coenzyme Q10 decreased pain intensity and cartilage degeneration and reduced MMPs, IL-6, IL-15, and nitrotyrosine levels [59].

## 5. The Effects of Antioxidants in KOA

### 5.1. Antioxidants and Their Metabolism

Considering that ROS overproduction leads to the emergence of oxidative stress, which has a role in the mechanisms of aging and the pathogenesis of many disorders among the geriatric population, restoring the redox balance is of essential importance. A reduction in the concentration of oxidant species may be achieved by using antioxidants. The human body controls the oxidative level through a series of endogenous antioxidants, enzymatical or nonenzymatical such as vitamins. Enzymes act specifically by eliminating ROS or synergically by catalyzing the regeneration of other antioxidants (for example, GR that regenerates GSH). In nature, there is a significant number of plant origin antioxidants, such as phenolic acids, flavonoids, carotenoids, and vitamins, which could be used to restore the oxidant–antioxidant balance [176]. In addition to natural antioxidants, synthetic ones have played an increasingly significant role in modern therapy. In this context, nanotechnologies have considerable implications in all areas of medicine, thus defining a new domain: nanomedicine. In this context, experts have synthesized magnetic vectors for the targeted delivery of antioxidants [177,178]. 

Based on the studies that revealed the helpful action of the antioxidants from the diet in relieving KOA symptoms, antioxidant-based therapies to restore the oxidative balance have been developed. Although there are publications that guarantee the beneficial effect of an adjuvant treatment with natural antioxidants, for their correct evaluation, it must be considered that the in vivo properties of exogenous antioxidants depend crucially on their stability. The key factor is the path of metabolic transformations, considering that the liver and the gastrointestinal tract generate metabolites with properties similar to, or different from, those of the original antioxidants. Therefore, it is vital to know the metabolic conversion of natural antioxidants to establish the opportunity of their application in adjuvant therapy. It should be noted that some natural antioxidants have reduced solubility in water and low bioavailability. The difficulty of passing through lipid membranes reduces the effectiveness of water-soluble antioxidants. All these characteristics condition the administration of antioxidants in high doses for satisfactory efficiency. Some authors noted that the treatment for KOA is recommended to be delivered directly into the synovial capsule, the location of the affected tissues being an advantage in this regard [179,180], although this practice sometimes showed a weak penetration of the cartilage [181]. Lately, nanotechnology has offered solutions to overcome some of the shortcomings discussed above. In recent years, functional metal oxide nanosystems have been developed to transport agents of natural [182] or synthetic antioxidants [178]. The most efficient nanosystems were made up of soft nanoparticles, such as polymeric micelles [183], nanoemulsions [184], and lipid vesicles [185]. 

Although studies show the molecular action mechanisms at the cellular level of exogenous antioxidants and their interactive or synergistic behavior toward endogenous antioxidants, we observed that all the discussed mechanisms, from the introduction of antioxidants into the body to the delivery to the targeted tissue, are still under debate, lacking concrete data. In this context, it is insufficiently documented how the antioxidant acts at the molecular level in the knee area when treatment of KOA with exogenous antioxidants is proposed. Consequently, it is complicated, or almost impossible, to establish a metabolic pathway from ingestion of exogenous antioxidants to the site of action represented by the knee. Given the existence of many publications in the field, we selected some antioxidants with beneficial effects in relieving OA, in order to analyze how they are metabolized in the human body and which are the key factors conditioning the pathology improvement [179]. 

Flavonoids found in vegetables in a glycosylated form are commonly listed as antioxidants in our diet. In an early metabolic stage, the flavonoids are deglycosylated [186,187], and then they are O-methylated at the catechol moiety in the liver and small intestine [187,188,189]. 

The changes of the antioxidant molecules can be even more drastic because, inside the colon, flavonoids can undergo destructive processes at the molecular level, transforming into smaller molecules with phenolic structure [188,190]. One of the most important flavonoid representatives, quercetin, causes the appearance of metabolic derivatives with antioxidant properties [191,192,193]. Morand et al. [194] showed that in plasma, most quercetin metabolites consist of quercetin-4’-*O*- sulphate, or 3’-O-methyl quercetin, and other derivatives with antioxidant properties [195]. The authors pointed out that their beneficial effects depend strongly on the regular consumption of plants containing polyphenols, as their effects decrease drastically in the first 20 h after ingestion [194,196]. The effects of quercetin on KOA changes were evaluated in animal studies and evidenced an increase in SOD concentration and tissue MMPs inhibitor of cartilage extracellular matrix degradation [197]. 

Resveratrol is a polyphenolic antioxidant found in peanuts, grapes, and teas. Resveratrol undergoes radical transformations in the human body in the gastrointestinal tract, leading to different products distributed in various organs. Springer and Moco [198] eloquently outline the complexity of resveratrol transformations. The compound undergoes sulfation and glucuronidation in the small intestine, while in the large intestine, it is metabolized by microorganisms resulting in dihydroresveratrol, lunularin, and 3,4’-dihydroxy-transstilbene. Resveratrol can also be metabolized in the liver and kidneys. Furthermore, the authors [198] have made an extensive assay, in which they put forward a list of resveratrol metabolites correlated with their presence in organs: trans-resveratrol-4’-O-glucuronides, trans-resveratrol-3-O-sulfate, dihydroresveratrol-glucuronide-sulfate, dihydroresveratrol-glucuronide-sulfate, and many others. 

Chlorogenic acid is an antioxidant found in larger amounts in coffee beans. Studies have shown that chlorogenic acid is stable in gastric fluids [199]. Although the metabolism of chlorogenic acid is extremely complex, the number of resulting derivatives being relatively high, its metabolic processes have been efficiently reviewed [200]. In this context, Farah and Duarte [201] proposed a concise scheme for the metabolic pathway of chlorogenic acids, highlighting the intermediates and final products with antioxidant properties, such as caffeic acid, vanilla, syringic acid, protocatechuic acid, and others, to illustrate the complex reaction pathways.

Oleuropein is a polyphenol found in olives and, to a greater extent, in olive leaves. Bock and coworkers quantified the bioavailability and oleuropein and hydroxytyrosol metabolism in human volunteers who were given olive leaf extract in various formulations [202]. MS analysis of urine and plasma showed different distributions in the two fluids depending on the oleuropein and hydroxytyrosol conjugates formulation (sulfated and glucuronidated) as part of the oleuropein transformation. The metabolite glucuronidated hydroxytyrosol is a free radical scavenger five times more efficient than hydroxytyrosol [203]. Oleuropein has shown high stability during digestion, while its glycosidated form reaches the colon and is transformed into a wide range of products due to microbial fermentation [204,205], mostly into its deglycosylated form and hydroxytyrosol [206]. Some urinary metabolic derivatives of oleuropein have been highlighted, such as oleuropein aglycone, hydroxytyrosol, homovanillyl alcohol, and an isomer of homovanillyl alcohol [207]. Although there are many published data, oleuropein is insufficiently studied in terms of metabolites found in various organs of the human body.

Catechins are polyphenols found in significant amounts in green tea (*Camellia sinensis)*, which have antioxidant properties. Catechins are stable enough to be excreted in feces, but most undergo conjugation reactions (glucuronide and sulfate formation) [208]. Some metabolic studies have shown that epigallocatechin-3-gallate can accumulate in the blood in a concentration high enough to exert antioxidant activity [209,210]. The metabolic products of catechins include glucuronides, sulfate conjugates, and methylated metabolites, most of which are metabolized in the liver and small intestine. Catechins undergo extensive transformations in the colon due to the action of microbes, leading to simple polyphenols [211]. 

Curcumin has been used in the study and treatment of clinical diseases due to its pharmacological properties [212]. Although oral curcumin supplementation has shown therapeutic efficacy in many clinical studies, its properties are counteracted by reduced bioavailability, low absorption, rapid metabolism, and elimination [213]. These shortcomings can be avoided using various drug formulations [179,213]. The curcumin metabolism occurs through oxidation, cleavage, conjugation, and reduction. It has been proved that curcumin is rapidly metabolized in the cell culture state and in vivo, mainly by reduction and conjugation [214]. Curcumin is conjugated to produce glucuronides and curcumin sulfates or is reduced to hexahydrocurcumin in the liver or intestines. Intraperitoneal or systemic administration reduces curcumin to tetrahydrocurcumin, hexahydrocurcumin, and octahydrocurcumin. Curcumin has limited stability and can degrade under physiological conditions. The degradation products were identified as trans-6-(40-hydroxy-30-methoxyphenyl)-2,4-dioxo-5-hexenal, ferulic acid, feruloyl methane, and vanillin. Some of the degrading curcumin products, such as ferulic acid or vanillin, may be associated with antioxidant activity [215]. 

In Table 1, the effects of the mentioned antioxidants in KOA context and the characteristics of the studies performed to determine their potentially chondroprotective and anti-inflammatory action are summarized.

### 5.2. Administration of Antioxidants in KOA

Nowadays, the data provided by in vitro and in vivo tests and the association between exogenous antioxidants and their metabolites in the generated therapeutic effects have made antioxidants a hot topic for research [226]. Despite the phenolic structure that allowed the elucidation of polyphenols’ redox mechanisms, the direct correlation of the antioxidant effect with the beneficial effects in KOA through their metabolites is extremely deficient. Better documented by in vivo studies of—KOA is the suite of effects that exogenous antioxidants can cause [216,217,219,220]. Consequently, the results show that exogenous antioxidants contribute to the control of pain in KOA, improving the physical function of the knee joints. All these observations substantiate the conclusion that exogenous antioxidants are good candidates for adjuvant treatment or as chemical structures that can inspire the design of new drugs.

Various ROS have been identified as relevant in KOA [227], thus being potential targets for prevention and/or therapeutic purposes. In this regard, a large amount of research has been performed, resulting in exciting findings, but without being systematized. 

As the ROS level is high in the context of KOA, it is recommended to follow a diet rich in fruits and vegetables with an antioxidant role or to take nutritional supplements that provide enzymes and vitamins, which can have a preventive effect [175]. Antioxidant effects resulting in benefits for the bone system have been attributed to sage extracts, which help osteoblastic differentiation [228], and to green tea [229].

Along with a protective role, a minimal reduction in pain intensity and improved joint function were associated with the consumption of strawberries, especially in obese patients with KOA [230]. Strawberries, similar to other berries [231], have antioxidant and anti-inflammatory properties due to their polyphenol content, and the effects have been supported by biochemical analysis of inflammatory markers, which exhibit lower concentrations when consuming strawberries for more extended periods of time [230]. In addition, the same effects accompanied by a reduction in edema and joint destruction are observed in the consumption of raspberries and blueberries [231,232] or of products containing resveratrol [233]. The effects of plant-derived resveratrol lead mainly to the reduction of the chondrocytes inflammation, which is produced by IL-1 [218], and limited KOA progression, as demonstrated in lab animal studies [234].

The health benefits of the antioxidant properties of flavonoids [235] and polyphenols [236] reside in their protective effects against cardiovascular diseases [237], DM, cancer [235], and in decreasing chronic inflammation [238], which encouraged the analysis of their effects in relation to KOA. Pomegranate juice has an antioxidant role and beneficial effects for KOA patients, improving pain scores and life quality. These health benefits have been demonstrated by biochemical analyses, which revealed decreased levels of MMPs and increased levels of GPx [239]. 

Sesame seeds also possess significant antioxidant activity, as they contain important amounts of phenolic compounds. Sesame seed products have antioxidant effects decreasing the serum values of MDA—a degradation product of lipid oxidation. The main action of sesame compounds is to reduce low-density lipoprotein (LDL)-cholesterol levels and lowering oxidative stress in patients with KOA [240]. Similar effects have been observed in overweight people taking food supplements with garlic [241] or with avocado oil and soy. Soybean and avocado oils contain campesterol, phytosterols, β-sitosterol, stigmasterol, fatty acids, and fat-soluble vitamins, which present anticatabolic properties, with a role in preventing cartilage destruction through the inhibition of the cell activity involved in this process and by stimulating the collagen production. This behavior reveals the chondro-protective role of such supplements, as their joint stiffness reduction and analgesic effects have been clinically demonstrated [242].

The Mediterranean diet has been much appreciated for its inclusion of many antioxidant-containing foods. In older adults on a Mediterranean diet, the symptomatic forms of KOA and pain were found to be much lower [243], while combining a diet that included extra-virgin olive oil with exercise was proved as a beneficial combination for cartilages and joints [244]. Olive oil is rich in active polyphenols, such as oleuropein, tyrosol, hydroxytyrosol, and oleocanthal, with important antioxidant and anti-inflammatory properties, being an important candidate in the prevention of degenerative changes [245]. The effects of oleuropein in KOA were also studied in association with curcumin or rutine (a glycoside found in citrus fruit). Less severe joint damage and decreased markers of inflammation and of cartilage degradation, such as aggrecans or prostaglandin E2, were correlated with their combined antioxidant action. However, no combination of these compounds could reduce serum-nitrated collagen levels (cartilage degradation marker) [246]. In this sense, it should be mentioned the extensive overview on putative antioxidant treatment strategies for KOA performed by Deligiannidou et al. [227].

Similar to chondroitin and glucosamine sulfate, some food supplements may also slow the progression of KOA [247]. These are glycosaminoglycans synthesized by chondrocytes and synovial cells, which are part of the synovial fluid and extracellular matrix. For exogenous intake, glucosamine sulfate can be extracted from crustaceans and chondroitin sulfate from animal cartilage [248]. A nonanimal chondroitin sulphate supplementation in obese patients with KOA also improved the knee function and inflammation [249]. When high concentrations of glucosamine sulphate are reached in the body, it acts by inhibiting IL-1, which participates in tissue destruction and inflammation. In vitro studies proved that glucosamine hydrochloride presents a pronounced scavenging effect on O_2_^•−^ and HO• [250]. The antioxidative properties of chondroitin have been studied, both in vitro and in vivo [251,252]. The proposed antioxidant mechanism involves the chelation of Fe^2+^ and cupric ion (Cu^2+^), which, as mentioned previously, are responsible for the generation of ROS. In addition, it was shown that chondroitin-4-sulfate holds higher antioxidant activity than chondroitin-6-sulfate [253]. The administration of glucosamine in certain formulas helps to better control pain, comparably to the effect of nonsteroidal anti-inflammatory medication, by slowing down the pathology progression and decreasing the need to perform arthroplasties, even a few years after stopping the treatment [254].

Certain herbs and even spices can have analgesic effects or improve joint functions, as highlighted by some reviewed data or multicenter studies [248,255]. Oral administration of such plants at specific doses led to decreased serum levels of MDA, cartilage oligomeric matrix protein, MPO, and IL-1β. On the other hand, it resulted in higher levels of SOD [132]. In addition to plant-based foods that have a beneficial role in balancing oxidative stress, antioxidant peptides from eggs act preventively in this regard by inactivating ROS, eliminating free radicals, reducing hydroperoxides, and chelating the pro-oxidative ions of transition metals [256]. 

To date, 710 antioxidant proteins are known; most of which (458) come from eukaryotes, 221 originate from bacteria, 28–from archaea, and three are viral [257]. Other perspectives for developing therapeutic options are offered by the improved heterologous expression of antioxidant enzymes derived from fungi, which are thermostable [258]. These have been called thermozymes and can be active at temperatures between 60 and 125 °C. In addition, they easily adapt to pH variations and have compact oligomers, increased stress resistance, and lifespan [259]. 

The protective mechanism against H_2_O_2_-induced ROS in human chondrocytes could be also provided by delphinidin. This antioxidant may have therapeutic potential for KOA by activating cytoprotective autophagy [260]. 

Moreover, the approach involving dietotherapy with foods having antioxidant effects and monitoring the redox status through biomarker levels is also important in the case of other pathologies, with high prevalence in geriatric patients [261]. 

## 6. Conclusions

KOA is a chronic multifactorial pathology, predominantly characteristic of older adults. It is a significant public health problem for which only symptomatic therapy is available. 

The current review emphasizes the importance of redox balance analysis. Thus, new individualized therapeutic perspectives are being developed to reduce oxidative stress levels in the human body, in correlation with the serum dosages of antioxidant and oxidative markers to maintain the redox balance.

Elucidating the oxidative stress generation mechanisms and the action mechanism of various antioxidants opens many possibilities in preventing and treating diseases associated with oxidative stress, especially in geriatric patients, with KOA being just one of the many pathologies associated with it.

This review highlights the significance of redox species and their imbalance, which can trigger oxidative stress, generating specific diseases. New therapeutic approaches to diseases associated with oxidative stress are also reviewed. It is worth mentioning that the specific benefits of an antioxidant-rich diet should be thoughtfully and systematically evaluated. It might provide an easy and economical way to treat KOA.

The reliability of the present review is based on the accuracy of the analyzed studies. There have been some reports of poorly controlled randomized clinical trials without any individual identification or isolation of the efficacious compounds, as well as a lack of uniformity in diagnosis. Such problems engender the limitations of the current review, stemming from the lack of pooled analysis or meta-analysis. The review emphasizes the need for high-quality randomized clinical studies using appropriate subjects with standardized outcome reports.

## Figures and Tables

**Table 1:** Antioxidants and Their Effect on KOA.

Antioxidant	Type of Study	Dosage/Time	Studied Samples/Analytes	Effect	Ref.
Quercitin	Animal model (rabbit) 48 subjects/ 16 controls	25 mg/kg 4 weeks	serum, synovial fluid, and synovial tissue SOD TIMP-1 MMP-13	↗ SOD and TIMP-1 ↘ MMP-13 ↗ KOA degeneration ↘ cartilage degradation	[197]
Olive Leaf Extract standardized for oleuropein content	In vivo, Randomized, double-blind, placebo-controlled, multi-centric trial 124 subjects/ mild disease	50 mg 6 months	serum Coll2-1NO2 MPO Osteocalcin	-no significant changes of inflammatory and cartilage remodeling biomarkers for low to moderate KOA -no significant changes of inflammatory and cartilage remodeling biomarker	[207]
Oleuropein	In vitro Human condrocytes	10, 50, 100 µM/2 h	chondrocytes culture COX-2 iNOS NO- PGE_2_ MMP-1, MMP-13	↘ NO production ↘ PGE_2_ production ↘ COX-2 ↘iNOS ↘MMP-1 ↘ MMP-13 ↘ ADAMTS-5 Suppresses NF-κB, MAPK activation	[216]
Curcumin	Animal model (mouse) synovial fluid of knee joints 30 subjects/ 15 controls	40 μmol/L/ 72 h	synovial fluid MMP-13	↘ MMP-13 -alleviate inflammation of osteoarthritis	[212]
Resveratrol	Animal model (rabbit)	50, 20, and 10 μmol/kg/ 2 weeks	cartilage sections (medial condyle) synovial fluid NO	↘ NO -the protective effect increased with increased dosage	[217]
Resveratrol	Clinical study 30 KOA patients human articular chondrocytes (harvested from osteoarthritis patients)	6.25–200 µM	chondrocytes culture TLR4 IL-1β products	-low concentrations (up to 25 µM) promoted cell proliferation, while a higher concentration (50–200 µM) had no effect -inhibition of TLR4/MyD88/ NF-κB signaling pathway	[218]
Chlorogenic acid	Animal model (rabbit) Local injection 16 subjects/ 4 controls	20 μM/ 6 weeks	anterior cruciate ligament transection MMP-1, MMP-3, MMP-13, TIMP-1	↗ TIMP-1 ↘ MMP-1 ↘ MMP-3 ↘ MMP-13	[219]
Green tea polyphenol epigallocatechin 3-gallate	In vitro Human OA cartilage	200 μM/24 h	OA cartilage COX-2 iNOS NO- PGE_2_	↘ COX-2 ↘ iNOS inhibit the production of NO and PGE_2_	[220]
Glucosamine chondroitin	Network meta-analysis on extremely different trials Bayesian model 10 trials/ 3803 patients	0.500 mg/3 time per day	pain intensity. -in minimal width of joint space	-no significant decrease in joint pain -no measurable impact on narrowing of joint space	[221,222]
Glucosamine sulfate (crystalline)	Long-term randomized, placebo-controlled trial 202 patients	1.5 g/day 3 years	pain intensity. change in minimal width of joint space	-significant reduction of joint structure changes -clinically relevant improvement of pain	[223]
Pomegranate extract (Flavonoids and polyphenols)	Animal model (rat)	0.50 mg/kg 28 days	chondrocyte culture Type II Collagenase PGE_2_ IL-1β	-inhibitory effects of punicalagin against Type II Collagenase, -inhibit the production of PGE_2_, ↘ IL-1β, -condroprotective effects: ↘ MMPs, ↘ Il, and ↘ PGs.	[224]
Rosmarinic acid (RosA water-soluble polyphenol, isolated from *Rosmarinus officinalis*.	Animal model (rat)	chondrocytes were exposed to RosA (10, 50, 100 µM) for 1 h	OA cartilage -chondrocytes culture IL-1β MMP-1 MMP-3 MMP-13 PGE_2_ iNOS COX-2	-inhibited the IL-1β-induced gene expression of MMP-1, MMP-3, and MMP-13 in a dose-dependent manner. ↘ MMP-1, MMP-3, and MMP-13 in chondrocytes, ↗ type II collagen, sulfated-proteoglycan, and PGE_2_ production, inhibited the protein expression of iNOS and COX-2 in chondrocytes.	[225]

ADAMTS-5: ADAM metallopeptidase with thrombospondin type 5 motif; Coll2-1NO2- biomarker of cartilage degradation; COX-2-cyclooxygenase-2; IL: interleukin; IL-1β-Interleukin-1 beta; iNOS: inducible nitric oxide synthase; MAPK: mitogen-activated protein kinase; MMPs: matrix metalloproteinases (1, 3 or 13); MKK3-mitogen-activated protein kinase-3; MyD88-Myeloid differentiation primary response protein; NO: nitric oxide; NF-κB: nuclear factor-kappa activated B cells; PGs—prostaglandines; PGE_2_-prostaglandin E_2_; RosA- Rosmarinic acid; SOD: superoxide dismutase; TIMP-1-tissue inhibitor of metalloproteinases-1; TLR4—Toll-like receptor 4. Both used arrows are symbols for increase and respetively for decrease.

## Abbreviations

4-HNE	4-hydroxynonenal
ATP	adenosine triphosphate
CAT	catalase
DM	diabetes mellitus
F2-IsoPs	isoprostanes
GC-MS	gas chromatography-mass spectrometry
GI-GPx	gastrointestinal GPx isoenzyme
GPx	glutathione peroxidase
GR	glutathione reductase
GSH	glutathione
GSSG	glutathione disulfide
GST	glutathione-S transferase
IKK	inhibitor of nuclear factor kappa-B kinase
IL	interleukin
IsoPs	isoprostanes
KL	radiological Kellgren–Lawrence classification system
KOA	knee osteoarthritis
LDL	low-density lipoprotein
MDA	malondialdehyde
MMPs	matrix metalloproteinases
MPO	myeloperoxidase
MS	mass spectrometry
mtDNA	mitochondrial DNA
NADPH	nicotinamide adenine dinucleotide phosphate
NEAC	nonenzymatic antioxidant capacity
NF-κB	nuclear factor kappa-light-chain-enhancer of activated B cells
NOX	nicotinamide adenine dinucleotide phosphate oxidase
OA	osteoarthritis
ox-LDL	oxidized low-density lipoprotein
Prx	peroxiredoxins
ROS	reactive oxygen species
SOD	superoxide dismutase enzyme
TAC	total antioxidants capacity
TNF-α	tumor necrosis factor alpha
